# Immunoglobulin G dose–exposure–efficacy response in patients with chronic inflammatory demyelinating polyradiculoneuropathy

**DOI:** 10.3389/fneur.2026.1911076

**Published:** 2026-09-24

**Authors:** Zhaoyang Li, Stefan Roepcke

**Affiliations:** 1 Clinical Pharmacology and Early Clinical Development, Takeda Development Center Americas, Inc., Cambridge, MA, United States; 2 Simulations Plus, Buffalo, NY, United States

**Keywords:** chronic inflammatory demyelinating polyradiculoneuropathy, dose-exposure-efficacy response, fSCIG 10%, hyaluronidase-facilitated subcutaneous immunoglobulin 10%, intravenous immunoglobulin 10%, IVIG, popPK–PD, population pharmacokinetic–pharmacodynamic model

## Abstract

**Introduction:**

Intravenous immunoglobulin (IVIG) 10% and hyaluronidase-facilitated subcutaneous immunoglobulin (fSCIG) 10% are both used as maintenance treatments for patients with chronic inflammatory demyelinating polyradiculoneuropathy (CIDP). Owing to the rarity of the condition, data regarding the relationship between dose, exposure, and clinical outcomes are sparse for these therapies. This analysis aimed to characterize the relationships between dose, exposure, and clinical efficacy of IVIG 10% and fSCIG 10% in CIDP.

**Materials and methods:**

Data were pooled from two phase 3 studies in patients with CIDP (NCT02549170; ADVANCE-CIDP 1) and multifocal motor neuropathy (NCT00666263). A sequential population pharmacokinetic (PK)–pharmacodynamic (PD) model was developed to characterize the PK–PD relationship between the PK of serum total immunoglobulin G (IgG) and, as the PD endpoint, the probability of increased adjusted Inflammatory Neuropathy Cause and Treatment (INCAT) disability score (higher scores indicate greater disability). The model was then used to simulate changes in total serum IgG concentrations and the PD endpoint for patients with CIDP. Dosing scenarios simulated were 6 months of IVIG 10% or 3 months of IVIG 10% followed by 9 months of fSCIG 10% at dosages of 0.4, 0.8, and 1.0 g/kg every 2 weeks, 1.0 and 2.0 g/kg every 3 weeks, and 0.4, 0.8, 1.0, and 2.0 g/kg every 4 weeks.

**Results:**

Our results showed that higher doses and more frequent IVIG/fSCIG 10% administration were associated with a lower mean probability of increased adjusted INCAT score (i.e., lower probability of relapse), remaining < 50% in all simulations except the 0.4 g/kg every 4 weeks dosage. The probability of increased adjusted INCAT score tended to be higher toward the end of the dosing interval for patients treated less frequently.

**Discussion:**

Higher and more frequent dosing may result in more consistent treatment efficacy in patients with CIDP.

## 1 Introduction

Chronic inflammatory demyelinating polyradiculoneuropathy (CIDP) is a rare neuronal disorder, with an estimated prevalence of 1.0–8.9 cases per 100,000 persons (1–3). In CIDP, there is an autoimmune-mediated response mounted against the myelin sheath of peripheral nerves, manifesting in progressive or relapsing proximal limb weakness and sensory loss, pain and fatigue, and significantly affected functional ability (1, 4).

The joint European Academy of Neurology/Peripheral Nerve Society guideline recommends treatment with corticosteroids or intravenous immunoglobulin (IVIG) as a first-line treatment option for CIDP (5). Short-term, high-dose IVIG may be used as induction treatment, in line with guidelines and according to patient response (5–7). In addition, most patients with CIDP will require maintenance therapy with IVIG, although the optimal maintenance dose and treatment schedule for IVIG are unknown (5). Another option for maintenance treatment for patients with CIDP is hyaluronidase-facilitated subcutaneous immunoglobulin (fSCIG) 10% (8, 9). fSCIG 10% comprises a dual-vial unit of immunoglobulin G (IgG) 10% and recombinant human hyaluronidase PH20, which depolymerizes hyaluronan in the extracellular matrix, transiently increasing the permeability of subcutaneous tissue to IgG (4, 8, 9). The recombinant human hyaluronidase PH20 component of fSCIG 10% therefore facilitates subcutaneous administration of IgG, in volumes equivalent to intravenous administration, over a short time period (4, 10). After fSCIG 10% is administered, serum IgG concentration gradually increases, reaching a lower peak than IVIG (median maximum concentration 15.5 g/L versus 21.9 g/L, respectively) while the median time to reach peak serum IgG levels is similar to conventional SCIG (approximately 5 days) (11). The safety profile of fSCIG 10% has been well-established in both patients with primary immunodeficiencies and CIDP (4, 11). Comparative analysis of safety between fSCIG 10% and IVIG has been carried out in patients with primary immunodeficiencies and is consistent with that of conventional SCIG, i.e., fewer systemic adverse reactions compared with IVIG (10–13); these comparative safety outcomes are expected to be similar for patients with CIDP. As with conventional SCIG, fSCIG 10% also allows for the convenience of self-administration at home (11).

CIDP is a more recent indication for fSCIG 10%, following the results of the phase 3, randomized, double-blind, multicenter, placebo-controlled ADVANCE-CIDP 1 trial (NCT02549170), which demonstrated the use of fSCIG 10% as maintenance treatment in patients with CIDP (4). fSCIG 10% is approved as maintenance therapy after stabilization with IVIG in adults and children/adolescents (aged 0–18 years) with CIDP in the EU, and in adults with CIDP in the United States (8, 9). The recommended doses of IVIG 10% and fSCIG 10% therapies vary based on body weight, and in practice, are individually adjusted to patient response (5–9).

In the larger body of evidence available for primary immunodeficiencies, serum IgG concentration is correlated with infection-related outcomes (14). An understanding of the relationship between dose, pharmacokinetics (PKs), and clinical outcomes enables serum IgG to be used as a marker for treatment effectiveness in a range of therapy areas (14, 15). Owing to the rarity of CIDP, data allowing assessment of the relationships between serum IgG concentration and clinical response following the administration of immunoglobulin therapies in affected patients are sparse. The understanding of these relationships is therefore limited in this population.

The use of population pharmacokinetic–pharmacodynamic (popPK–PD) models, which have become integral in drug development and regulatory review, has enabled more in-depth understanding of PK–PD relationships in rare diseases when data are sparse (16, 17). Recent publications reported the successful use of popPK and popPK–PD modeling to describe the relationship between dose and exposure, as well as dose–exposure–efficacy response, during treatment with IVIG 10% in patients with multifocal motor neuropathy (MMN) (15, 18). Both MMN and CIDP are immune–mediated peripheral neuropathies that respond to immunoglobulin therapy, which is indicated to improve neuromuscular disability and impairment with similar dosing regimen (6). This is the first popPK–PD modeling study describing the interplay between fSCIG 10% dose, total IgG serum concentration, and clinical efficacy in patients with CIDP, and it will help to provide insights into optimal dosing strategies for this population. The pooling of PK data from limited samples of patients with MMN and patients with CIDP was considered in an attempt to increase the statistical power for more accurate parameter estimates.

The main aims of this analysis were to characterize the popPK–PD relationships of fSCIG 10% and IVIG 10% in patients with CIDP and to provide insights into the interplay between IgG dose, exposure, and clinical response.

## 2 Materials and methods

### 2.1 Data sources and handling

PK and clinical response data were pooled from two phase 3, multicenter studies, for which full details of methodology and clinical results have previously been published (4, 19). In brief, ADVANCE-CIDP 1 (Study 1) evaluated fSCIG 10% efficacy, safety, and tolerability in patients with probable or definite CIDP (*n* = 132) who were receiving a stable dosing regimen of IVIG 10% (equivalent to a cumulative monthly dose of 0.4–2.4 g/kg) for ≥ 12 weeks before screening (4). The study consisted of two treatment epochs. In Epoch 1, following a baseline period of ≤ 8 weeks, patients were randomized (1:1) to receive either fSCIG 10% (administered at an equivalent dose to that of their previous IVIG 10%) or placebo with recombinant human hyaluronidase PH20 for 6 months. In Epoch 2 (also lasting 6 months), patients who relapsed during Epoch 1 received open-label IVIG 10% to restore functional ability (4). An induction dose of IVIG 10% 2.0 g/kg was administered over 2–5 consecutive days, followed by maintenance infusions at the same monthly equivalent to pre-randomization IVIG 10%. The primary endpoint was relapse rate, defined as the proportion of patients who experienced an increase in adjusted Inflammatory Neuropathy Cause and Treatment (INCAT) disability score of ≥ 1, relative to baseline, in two consecutive assessments within 7 days of each other (4). INCAT scores (scored 0–10) consist of upper and lower limb components, with higher scores indicating greater disability (4). For adjusted INCAT scores, changes in upper limb score from 0 to 1 or from 1 to 0 (normal to minor symptoms or vice versa) were excluded (4). Throughout the study, 2–5 serum trough IgG samples per patient were collected for PK analysis in both epochs.

Study 2 (NCT00666263) was a randomized, double-blind, placebo-controlled crossover trial, evaluating IVIG 10% as treatment in adults with definite or probable MMN (*n* = 44) (19). Eligible patients were receiving a stable dosage of IVIG 10%, 0.4–2.0 g/kg every 2–5 weeks for ≥ 3 months at enrollment. Patients received open-label IVIG 10% in an initial 12-week clinical stabilization period, followed by a double-blind crossover period in which patients were randomized 1:1 into one of two treatment sequences: 12 weeks of IVIG 10% or placebo followed by 12 weeks of placebo or IVIG 10%. Each double-blind crossover period was proceeded by 12 weeks of open-label IVIG 10% for stabilization, which patients were permitted to enter into early (accelerated switch) if they experienced significant deterioration. The dose range of IVIG 10% was 0.4–2.0 g/kg/infusion cycle, divided over 1–5 consecutive days, every 2, 3, or 4 weeks (19). Serum total IgG concentration (assessed at screening, before dose administration at the beginning of each stabilization/crossover period, at the end of the final stabilization period, and at study end) was used for PK analysis.

Individual patient data, including information on PK sampling, treatment assignment, and demographics, were extracted from both studies and included in the data set used for the PK model development; adjusted INCAT score assessments were included from Study 1 for development of the PK–PD model. Informed consent was obtained in both studies. This modeling study used pre-existing anonymized data from previous studies; therefore, no ethics approval or consent for publication was required.

### 2.2 Data analysis

#### 2.2.1 PopPK–PD model development

The sequential popPK–PD model was developed for serum total IgG PK and the adjusted INCAT score using nonlinear mixed effects modeling with NONMEM^®^, version 7.3 (ICON, Hanover, NH, United States) (20). A preliminary exploratory analysis was used to assess trends and identify possible outlier values prior to model development, and to aid understanding of the informational content of the data set with respect to efficacy (PD) parameters. The parameters evaluated as potential PD endpoints were grip strength of the more and less affected hands, adjusted INCAT score, Rasch-Built Overall Disability Scale raw summed and centile metric scores, and Medical Research Council sum score. The general procedure that was followed included base structural model development, evaluation of covariate effects, and model evaluation using prediction-corrected visual predictive checks. A one-compartment PK model was used, with between-patient variability of the endogenous component of IgG and standard quarter-power scaling of PK structural parameters and the individual Bayesian estimates from the popPK analysis as fixed input. The IgG concentrations predicted by the PK model were linked to the probability of relapse per unit of time in terms of a binary response model for increases in adjusted INCAT score. A systematic covariate analysis was then performed to identify potential covariates affecting the PK–PD of fSCIG 10%.

#### 2.2.2 Simulations

The final sequential popPK–PD model was used to simulate changes in total serum IgG concentrations and the probability of increased adjusted INCAT score for patients with CIDP. For each treatment scenario, a virtual population of 2,500 patients was created by resampling from the observed body weights of the patients with CIDP relapse (*n* = 28). The treatment scenarios varied by dose levels and dosing frequencies. The scenarios fall under two categories: (1) 24 weeks of continuous IVIG 10%, or (2) 12 weeks of IVIG 10% followed by fSCIG 10% ramp-up dosing (Table 1), and then 36 weeks of fSCIG 10% maintenance therapy. The simulated scenarios include the following combinations of dose and dosing frequency: 0.4, 0.8, and 1.0 g/kg administered every 2 weeks, 1.0 and 2.0 g/kg every 3 weeks, and 0.4, 0.8, 1.0, and 2.0 g/kg every 4 weeks. Summary profiles of the simulated IgG concentrations and probability of increased adjusted INCAT scores for each dosing scenario were plotted, assuming typical values for popPK–PD model parameters and median body weight values.

**Table 1:** fSCIG 10% dose ramp-up schedule, depending on dosing interval during the maintenance phase.

Maintenance treatment interval	Week							
	1	2	3	4	5	6	7	8
Q2W	1/2 × D	1/2 × D	D	–	–	–	–	–
Q3W	1/3 × D	1/3 × D	2/3 × D	–	D	–	–	–
Q4W	1/4 × D	1/4 × D	1/2 × D	–	3/4 × D	–	–	D

D, first full fSCIG 10% dose after ramp-up to be continued as maintenance dose; fSCIG, hyaluronidase-facilitated subcutaneous immunoglobulin; Q2W, every 2 weeks; Q3W, every 3 weeks; Q4W, every 4 weeks.

## 3 Results

### 3.1 Analysis data set and patient population

The final analysis data set consisted of 486 serum IgG concentrations from 131 patients with CIDP in Study 1, and 310 serum IgG concentrations from 44 patients with MMN in Study 2. The mean (standard deviation) age of patients included in the data set was 53.8 (13.0) years; 60.2% were male, and 92.6% were White. The mean (standard deviation) weight and lean body mass of patients were 82.5 (17.7) kg and 54.4 (9.1) kg, respectively. Baseline clinical characteristics and covariates are summarized in Supplementary Table S1. The analysis data set included 786 adjusted INCAT score records from 130 patients (124 patients after randomization) with CIDP in Study 1.

### 3.2 Exploratory analysis of correlation between PK and adjusted INCAT scores

At Study 1 baseline, adjusted INCAT scores ranged from 1 to 7, and there were no obvious correlations between dose–exposure measures (area under the concentration–time curve and trough concentration) and adjusted INCAT score (Figure 1). Given that patients were required to have received a stable dose of IVIG for ≥ 12 weeks before screening but could have INCAT disability scores ranging 0–7 at baseline, a correlation would not be expected in these patients at baseline. Adjusted INCAT scores between 1 and 8 were observed throughout Study 1. During Epoch 1 (6 months of either fSCIG 10% or placebo), the mean (standard deviation) change in adjusted INCAT score from baseline was −0.236 (0.666) in the fSCIG 10% group, compared with 0.261 (1.2) in the placebo group (Figure 2). During the study, 28/132 patients (21.2%) experienced relapse, of which 22 patients were receiving placebo and 6 patients were treated with fSCIG 10%. There was no difference in the baseline adjusted INCAT scores of patients who experienced relapse during placebo treatment versus those who did not. However, on average, IgG exposure at baseline was higher for patients who experienced relapse than for those who did not (Figure 3), as were weight-based monthly doses. Of the 28 patients who experienced relapse, 21 entered Epoch 2 (6 months of IVIG 10%), resulting in improvement of adjusted INCAT score in 19/21 patients; these data were included in the data set.

**Figure 1:**
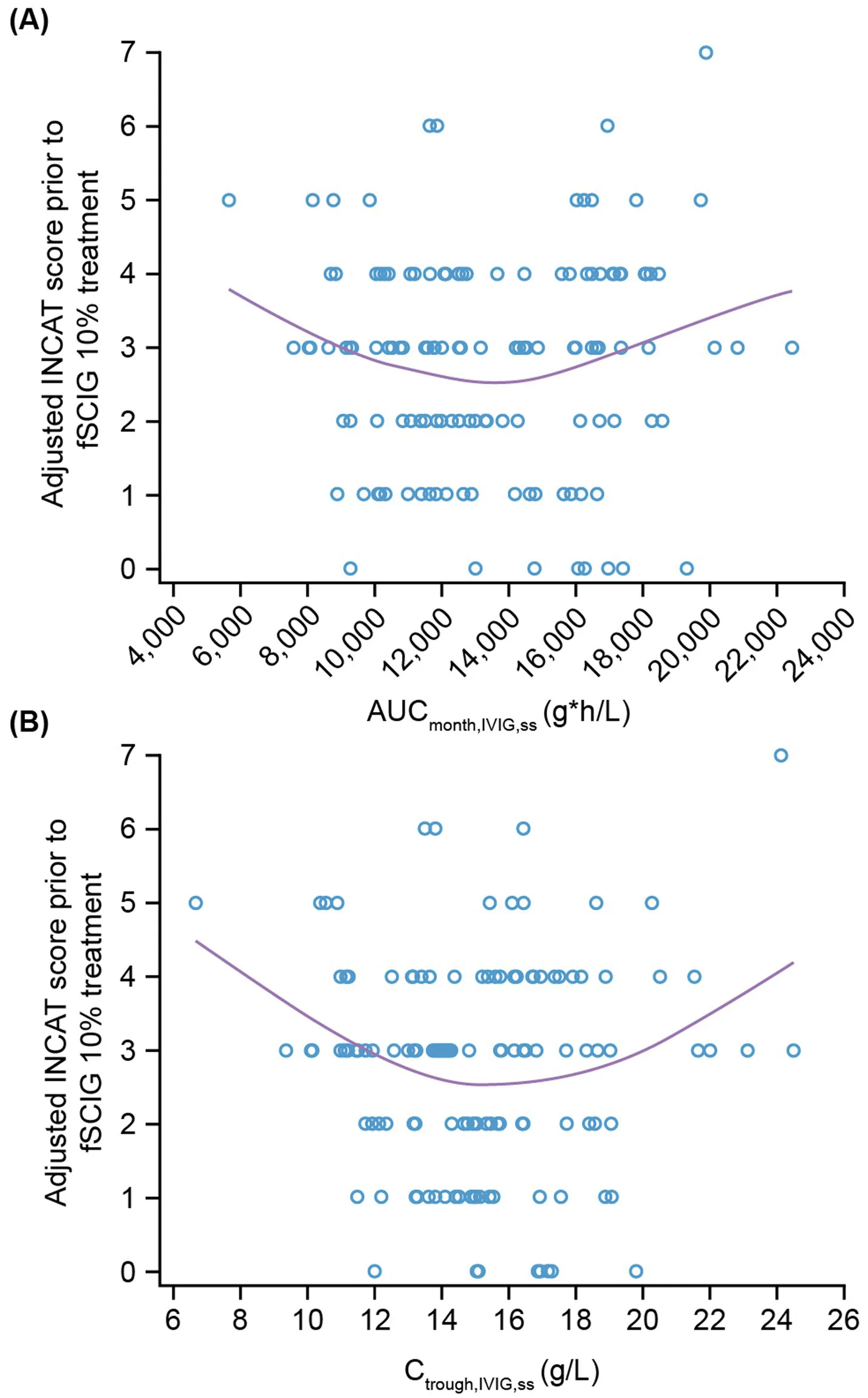
Relationship between adjusted INCAT score and dose–exposure measures (AUC and C_trough,IVIG,ss_), with no obvious correlations being shown. **(A)** INCAT score versus monthly AUC at baseline (stabilized on IVIG 10%). **(B)** INCAT score versus C_trough,IVIG,ss_. The line represents a smoothing spline fit to the data. AUC, area under the concentration–time curve; AUC_month,IVIG,ss_, AUC over 1 month at steady state during treatment with IVIG 10%; C_trough,IVIG,ss_, trough immunoglobulin G concentration of IVIG 10% at steady state; fSCIG, hyaluronidase-facilitated subcutaneous immunoglobulin; INCAT, inflammatory Neuropathy Cause and Treatment; IVIG, intravenous immunoglobulin.

**Figure 2:**
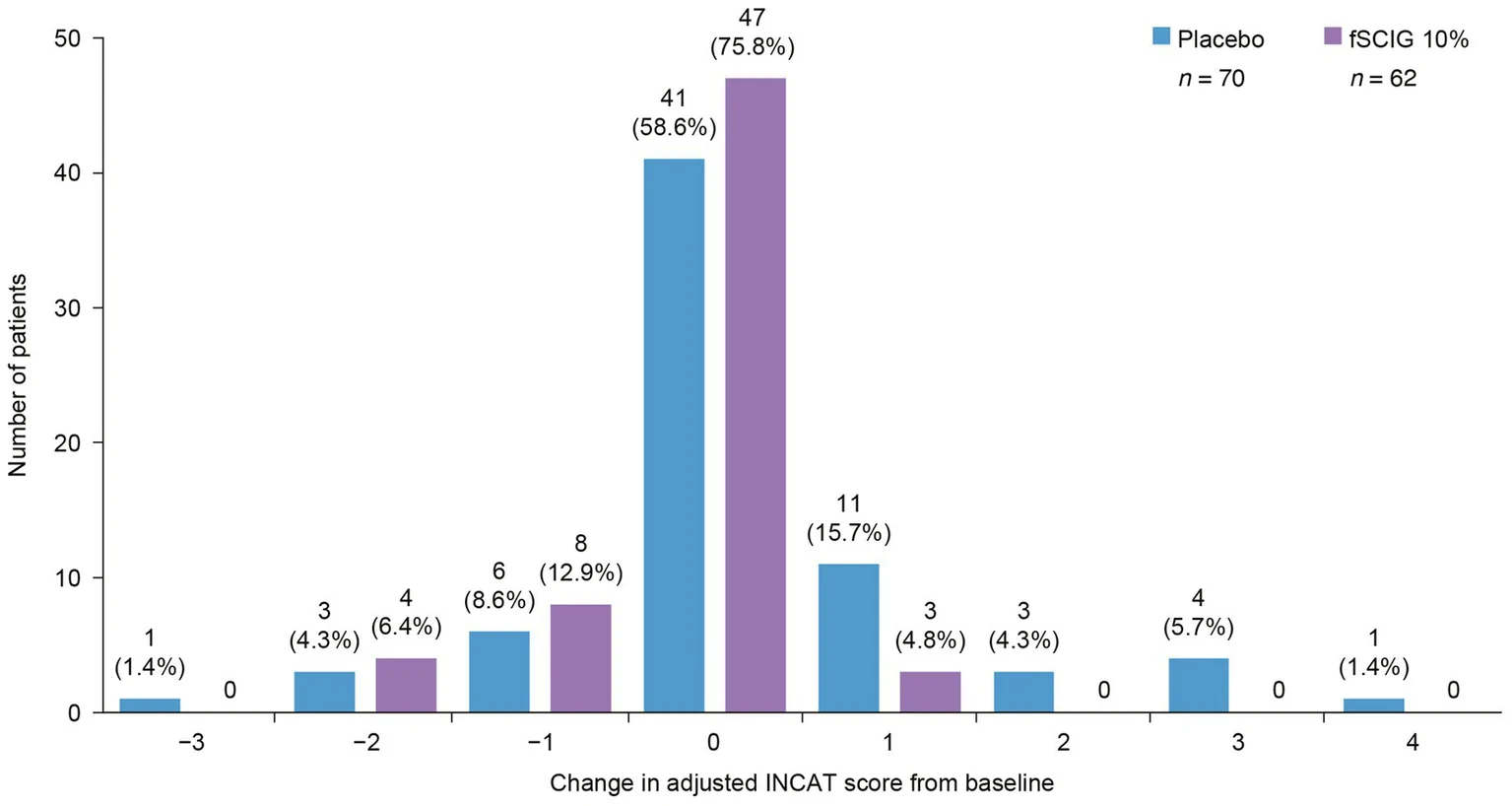
Change in adjusted INCAT score from baseline in patients with CIDP receiving fSCIG 10% or placebo in Study 1 Epoch 1. Change in INCAT score is calculated as the difference between baseline score and score observed at the final observation in Epoch 1. CIDP, chronic inflammatory demyelinating polyradiculoneuropathy; fSCIG, hyaluronidase-facilitated subcutaneous immunoglobulin; INCAT, Inflammatory Neuropathy Cause and Treatment.

**Figure 3:**
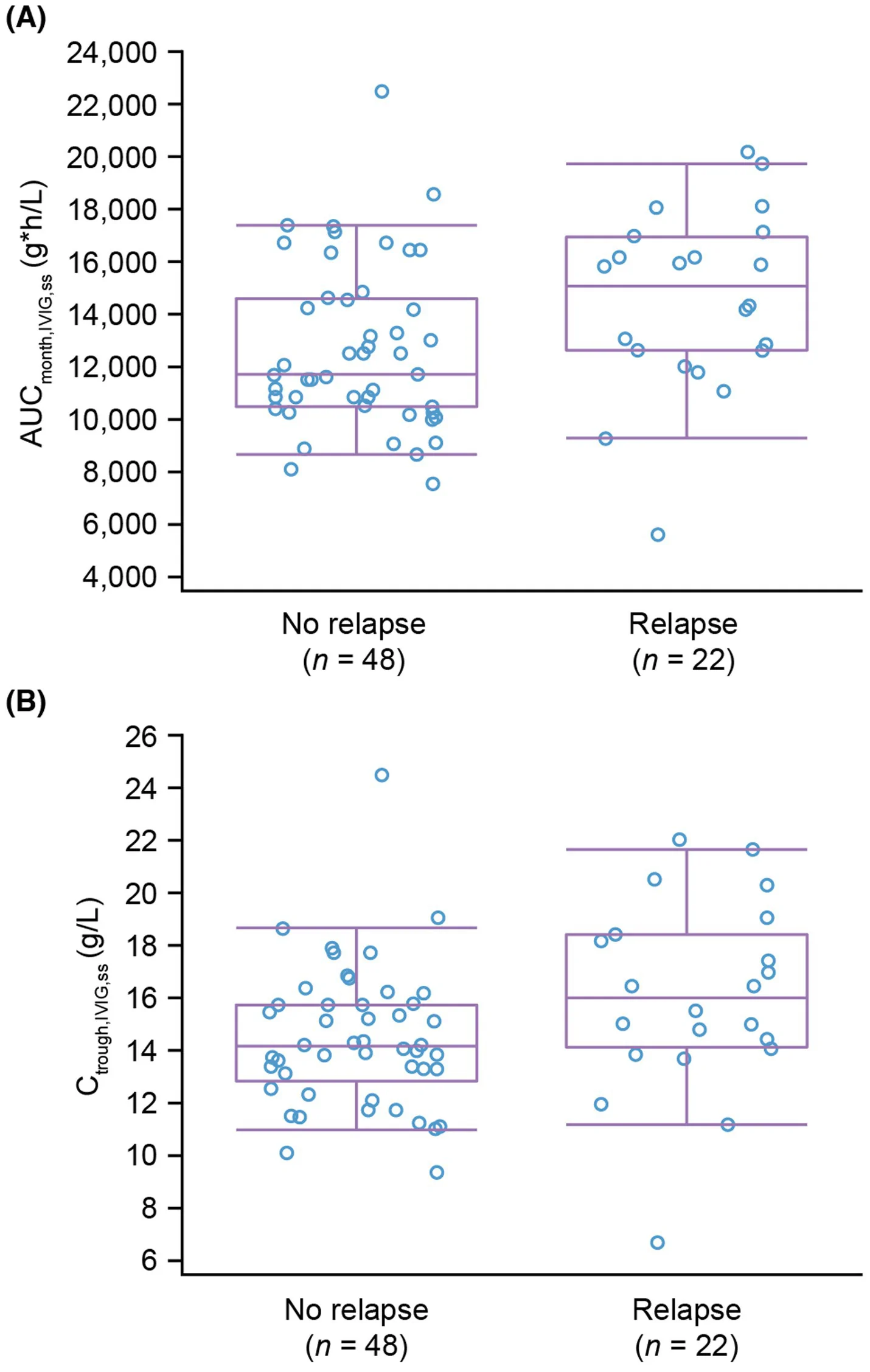
Comparison of pre-fSCIG 10% exposure parameters between patients with CIDP receiving placebo who experienced relapse and those who did not in Study 1. **(A)** AUC_month,IVIG,ss_. **(B)** C_trough,IVIG,ss_. Boxes are 25th, 50th, and 75th percentiles. Whiskers are 5th and 95th percentiles. AUC_month,IVIG,ss_, area under the concentration–time curve over 1 month at steady state during treatment with intravenous immunoglobulin 10% (before randomization); CIDP, chronic inflammatory demyelinating polyradiculoneuropathy; C_trough,IVIG,ss_, trough immunoglobulin G concentration of intravenous immunoglobulin 10% at steady state; fSCIG, hyaluronidase-facilitated subcutaneous immunoglobulin.

### 3.3 PopPK–PD model development and evaluation

The final model was a sequential popPK–PD model consisting of a one-compartment PK part for serum total IgG and a PD part for the probability of increased adjusted INCAT score as the measure of response (Supplementary Figure S1; Supplementary Table S2). This model adequately described relationships between fSCIG 10% and IVIG 10% dose regimens, serum total IgG concentrations, and the probability of experiencing an increase in adjusted INCAT score relative to baseline. Potential covariates, including disease classification, were tested systematically (univariate analysis); however, none of them explained a statistically significant portion of interindividual variability (Supplementary Table S1). Therefore, no additional covariate was included in the final sequential popPK–PD model.

### 3.4 Simulations

Simulated IgG levels for patients with CIDP receiving fSCIG 10% and IVIG 10% as part of different dosing scenarios are presented in Figure 4. Trough IgG levels during fSCIG 10% treatment after switching from IVIG 10% increased with dose and were marginally lower than during IVIG 10% therapy. The mean probability of increased adjusted INCAT score remained < 50% for both fSCIG 10% and IVIG 10%, except at the simulated lowest dose given at the longest interval (0.4 g/kg every 4 weeks) (Figure 5). Simulated higher doses and/or more frequent treatment administration led to a lower probability of increased adjusted INCAT score for both IVIG 10% (Table 2) and fSCIG 10% (Table 3). With the most frequent dosing (i.e., every-2-week dosing), the steady-state median (5th, 95th percentile) probability of increased adjusted INCAT score at the end of the treatment interval at a 1.0 g/kg dose was 0.004 (< 0.001, 0.529) for IVIG 10% and 0.009 (< 0.001, 0.584) for fSCIG 10%. With every-4-week dosing, at a 0.4 g/kg dose, the median (5th, 95th percentile) probability of increased adjusted INCAT score was 0.621 (0.007, 0.779) for IVIG 10% and 0.637 (0.017, 0.78) for fSCIG 10%.

**Figure 4:**
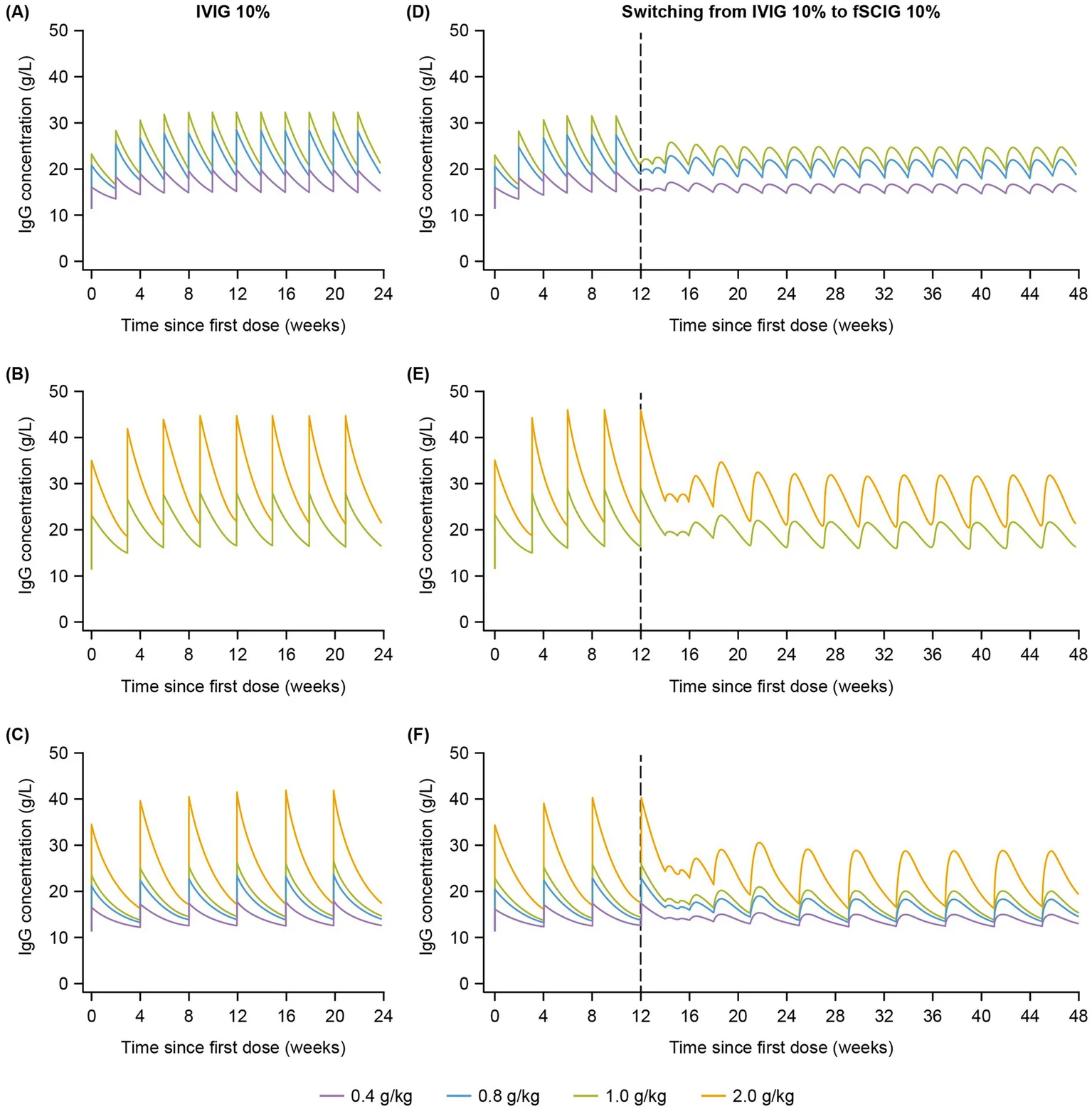
Simulated mean serum IgG concentration profiles for IVIG 10% or fSCIG 10% at different dosing frequencies. **(A)** IVIG 10% every 2 weeks. **(B)** IVIG 10% every 3 weeks. **(C)** IVIG 10% every 4 weeks. **(D)** fSCIG 10% every 2 weeks. **(E)** fSCIG 10% every 3 weeks. **(F)** fSCIG 10% every 4 weeks, after switching from IVIG 10%. fSCIG, hyaluronidase-facilitated subcutaneous immunoglobulin; IgG, immunoglobulin G; IVIG, intravenous immunoglobulin.

**Figure 5:**
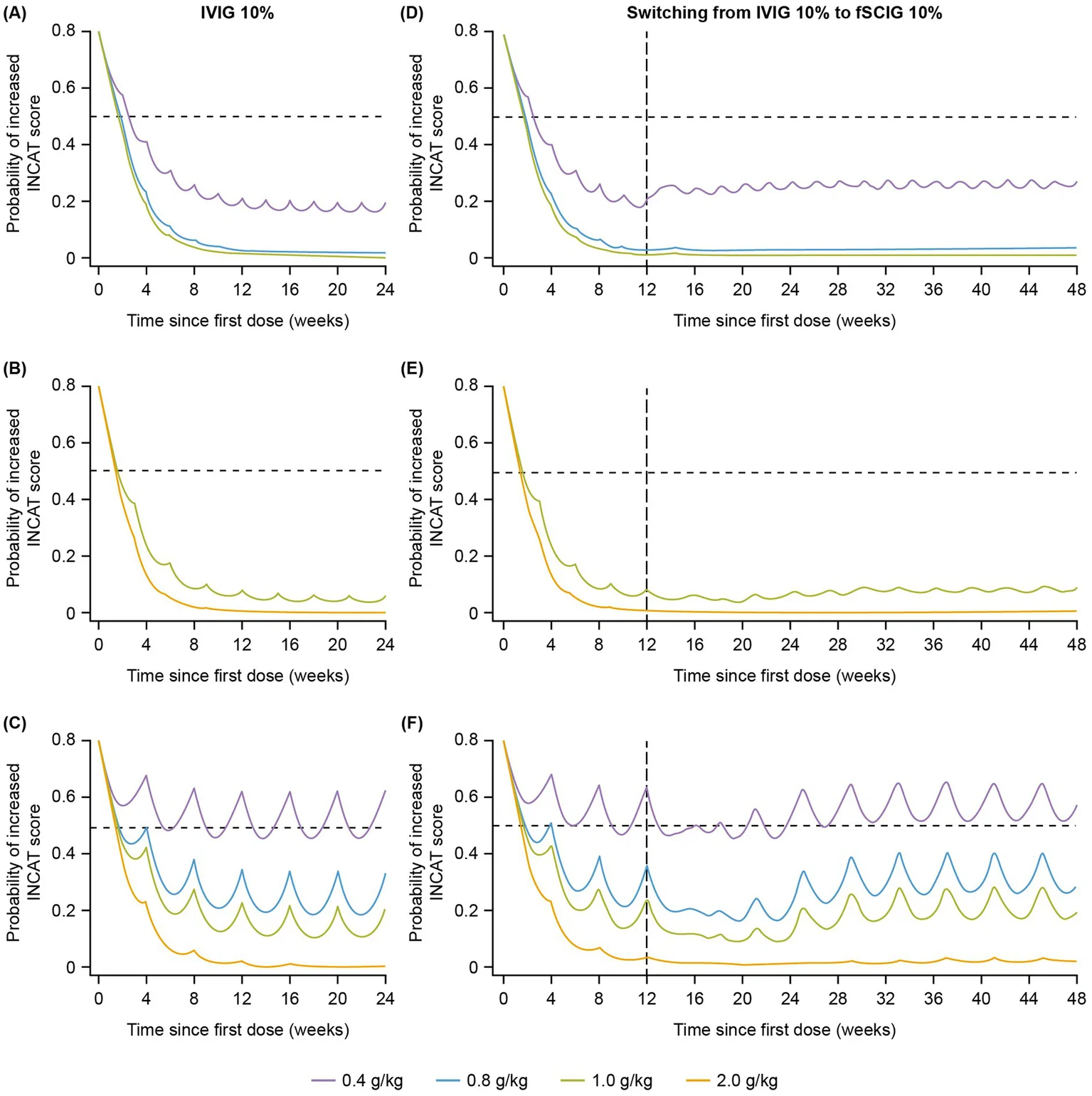
Simulated mean probability of increased adjusted INCAT score for IVIG 10% and fSCIG 10% at different dosing frequencies. **(A)** IVIG 10% every 2 weeks. **(B)** IVIG 10% every 3 weeks. **(C)** IVIG 10% every 4 weeks. **(D)** fSCIG 10% every 2 weeks. **(E)** fSCIG 10% every 3 weeks. **(F)** fSCIG 10% every 4 weeks, after switching from IVIG 10%. Horizontal dashed lines denote 0.5 probability. Vertical dashed lines denote the switch from IVIG 10% to the start of the fSCIG 10% ramp-up period. fSCIG, hyaluronidase-facilitated subcutaneous immunoglobulin; INCAT, Inflammatory Neuropathy Cause and Treatment; IVIG, intravenous immunoglobulin. This figure is adapted from a poster presented by the authors at the Annual Meeting of the American Association of Neuromuscular and Electrodiagnostic Medicine (AANEM) 2024, October 15–18, 2024.

**Table 2:** Simulated probabilities of increased adjusted INCAT scores for IVIG 10% at the end of the treatment interval.

Treatment interval	IVIG 10% dose, g/kg			
	0.4	0.8	1.0	2.0
Q2W				
Mean (SD)	0.273 (0.26)	0.135 (0.204)	0.096 (0.175)	–
Median (5th, 95th percentile)	0.205 (< 0.001, 0.724)	0.016 (< 0.001, 0.611)	0.004 (< 0.001, 0.529)	–
Q3W				
Mean (SD)	–	–	0.191 (0.239)	0.078 (0.159)
Median (5th, 95th percentile)	–	–	0.0555 (< 0.001, 0.686)	0.001 (< 0.001, 0.484)
Q4W				
Mean (SD)	0.515 (0.26)	0.354 (0.28)	0.291 (0.27)	0.143 (0.22)
Median (5th, 95th percentile)	0.621 (0.007, 0.779)	0.358 (< 0.001, 0.756)	0.23 (< 0.001, 0.737)	0.012 (< 0.001, 0.655)

INCAT, Inflammatory Neuropathy Cause and Treatment; IVIG, intravenous immunoglobulin; Q2W, every 2 weeks; Q3W, every 3 weeks; Q4W, every 4 weeks; SD, standard deviation.

**Table 3:** Simulated probabilities of increased adjusted INCAT scores for fSCIG 10% at the end of the treatment interval.

Dosing interval	fSCIG 10% dose, g/kg			
	0.4	0.8	1.0	2.0
Q2W				
Mean (SD)	0.314 (0.264)	0.163 (0.219)	0.116 (0.19)	–
Median (5th, 95th percentile)	0.295 (< 0.001, 0.729)	0.037 (< 0.001, 0.654)	0.009 (< 0.001, 0.584)	–
Q3W				
Mean (SD)	–	–	0.213 (0.245)	0.086 (0.169)
Median (5th, 95th percentile)	–	–	0.092 (< 0.001, 0.687)	0.001 (< 0.001, 0.525)
Q4W				
Mean (SD)	0.536 (0.249)	0.365 (0.28)	0.314 (0.276)	0.167 (0.229)
Median (5th, 95th percentile)	0.637 (0.017, 0.78)	0.378 (< 0.001, 0.759)	0.271 (< 0.001, 0.746)	0.029 (< 0.001, 0.658)

fSCIG, hyaluronidase-facilitated subcutaneous immunoglobulin; INCAT, Inflammatory Neuropathy Cause and Treatment; Q2W, every 2 weeks; Q3W, every 3 weeks; Q4W, every 4 weeks; SD, standard deviation.

## 4 Discussion

CIDP is a rare condition, and evidence for dose–exposure–response relationships, which are beneficial for guiding dosing and study design, is lacking. This popPK–PD model was developed to characterize the relationships between dose, serum total IgG level, and probability of increased adjusted INCAT score in patients with CIDP receiving IVIG 10% or fSCIG 10% as maintenance therapy.

As part of the exploratory analyses of dose–serum IgG exposure correlation and adjusted INCAT score conducted during model development, it was noted that median IgG exposures at baseline were higher in patients who experienced relapse during placebo treatment than in those who did not, while the weight-based monthly dose was also higher. This may indicate that higher therapy doses were needed for patients experiencing worse symptoms in relapse. However, the data should be interpreted with caution, given that the total number of patients (*N* = 28) was small and that there was a high degree of overlap in the data for patients who experienced relapse and those who did not. PK–PD model parameters relied mainly on data from the placebo group who received stable IVIG treatment prior to Study 1; however, the data set was restricted to patients who experienced relapse in Study 1, where the majority of patients receiving placebo did not relapse and did not enter Epoch 2 of Study 1. In addition, no difference in patient- or treatment-related characteristics between those who relapsed and those who did not was identified; this model is therefore most appropriate for patients who experience relapse when treatment is interrupted. It was recognized during model development that, for most patients, INCAT scores either did not change or changed by only ± 1 point from baseline throughout the study period, presenting significant challenges to the model if INCAT score is used directly as the PD endpoint. The popPK–PD model was therefore constructed to estimate the probability of observing ≥ 1-point change in adjusted INCAT score from baseline.

Model evaluation demonstrated that the final sequential popPK–PD model for binary response data adequately described PK–PD relationships for IVIG 10% and fSCIG 10%. The simulations based on this model illustrated that higher doses and/or more frequent administration of IVIG 10% or fSCIG 10% as maintenance treatment resulted in lower mean probability of increased adjusted INCAT score (indicating worse disability), which remained < 50% in all simulations, except at the lowest dose and longest dosing interval for both therapies. The probability of increased adjusted INCAT score tended to be higher toward the end of the dosing interval for patients treated less frequently; increased dosing frequency may help to maintain more consistent IgG levels. This is consistent with findings from a popPK modeling analysis, which simulated steady-state serum IgG trough concentrations after various IVIG 10% dosing regimens with 2-, 3-, and 4-week intervals in patients with MMN; the analysis demonstrated lower pre-dose trough IgG levels and larger fluctuations with every-4-week dosing compared with shorter dosing intervals (15). The findings of this analysis support the results of the ADVANCE-CIDP 1 trial, which demonstrated beneficial efficacy outcomes for fSCIG 10% as maintenance therapy for patients with CIDP (4). They also offer additional guidance for clinicians seeking to optimize dosing regimens for affected patients.

We acknowledge the limitations of this analysis. PK data were pooled from two studies that differed in study design and patient population. However, the study populations were considered similar enough for PK findings to apply to both patients with CIDP and those with MMN. Serum IgG concentrations and PD parameter observations were not available for treatment-naive patients, and total IgG concentrations at the baselines of the studies were measured after IVIG 10% or fSCIG 10% administration; thus no distinction between endogenous and exogenously administered IgG in the blood sample was possible. However, individual endogenous baseline levels were estimated based on the model. Future studies may evaluate other biomarkers for CIDP, but at present, sufficient data for a robust PK–PD analysis are lacking. Adjusted INCAT scores were only measured in patients with CIDP and did not change from baseline for many patients throughout the study. In addition, many of the patients in the placebo group did not relapse; therefore, the data utilized for model development and parameter estimation were limited to 28 patients, which may affect the generalizability. However, this number of patients applies to PK–PD parameters only, the total number of patients (*N* = 175) is sufficient for a PK analysis, and pooling allowed better parameter estimates.

Owing to the rarity of this disease, considerations for minimizing patient burden, and the consequently limited PK data, modeling allows for evidence generation for dosing strategies and helps to inform future clinical trial design. This popPK–PD model provided insights into the interplay between dose, total IgG exposure, and clinical efficacy in patients with CIDP. Additionally, we presented simulations of total IgG serum concentrations and the corresponding probability of increases in adjusted INCAT score for various dosing regimen scenarios for fSCIG 10% and IVIG 10% in patients with CIDP. The findings of this analysis demonstrate that higher and more frequent dosing may result in a more consistent treatment efficacy in patients with CIDP.

## Data Availability

The data that support the findings of this study are available from the corresponding author upon reasonable request. Requests to access these data sets should be directed to Zhaoyang Li, zhaoyang.li@takeda.com.
