# Supplementary material for: Immunoglobulin G dose–exposure–efficacy response in patients with chronic inflammatory demyelinating polyradiculoneuropathy
Source: Front Neurol. 2026 Sep 24;17:1911076. doi: 10.3389/fneur.2026.1911076 (PMC13647578; doi:10.3389/fneur.2026.1911076)
Supplement: Supplementary file 1 [file Supplementary_file_1.DOCX]

# Supplementary figures

**SUPPLEMENTARY FIGURE S1**
Schema of the popPK–PD model. B, logit of the probability of an increased adjusted INCAT score in the absence of drug; C50, difference between the IgG concentration and latent model-derived parameter of endogenous IgG concentration, at which the pharmacological effect is half-maximal; E_max_, maximum pharmacologic effect; F, bioavailability fraction; fSCIG, hyaluronidase-facilitated subcutaneous immunoglobulin; IgG, immunoglobulin G; INCAT, inflammatory neuropathy cause and treatment; IVIG, intravenous immunoglobulin; K_A_, rate of absorption; K_EL_, IgG elimination rate; K_IN_, endogenous production rate of IgG; KR_EL_, rate constant of response decrease; KR_IN_, rate constant of response maintenance; Res, response compartment; popPK–PD, population pharmacokinetic–pharmacodynamic; SC, subcutaneous; V_1_, central volume of distribution of IgG.

# Supplementary tables

**SUPPLEMENTARY TABLE S1**	Summary of patient demographics and covariates.

| **Patient characteristic** | **Study 1 (CIDP)**  **(NCT02549170)**  **(*n* = 132)** | **Study 2 (MMN)**  **(NCT00666263)**  **(*n* = 44)** | **Overall**  **(*n* = 176)** |
| --- | --- | --- | --- |
| **Age, years** |  |  |  |
| Mean (SD) | 54.4 (13.8) | 51.7 (10.3) | 53.8 (13.0) |
| Median (min, max) | 56.0 (19, 86) | 52.0 (31, 72) | 54.0 (19, 86) |
| **Sex, *n* (%)** |  |  |  |
| Male | 74 (56.1) | 32 (72.7) | 106 (60.2) |
| Female | 58 (43.9) | 12 (27.3) | 70 (39.8) |
| **Race, *n* (%)** |  |  |  |
| White | 122 (92.4) | 41 (93.2) | 163 (92.6) |
| Black | 0 (0.0) | 2 (4.5) | 2 (1.1) |
| American  Indian/Alaska Native | 3 (2.3) | 0 (0.0) | 3 (1.7) |
| Multiple | 1 (0.8) | 0 (0.0) | 1 (0.6) |
| Not reported | 6 (4.5) | 1 (2.3) | 7 (4.0) |
| **Ethnicity, *n* (%)** |  |  |  |
| Not Hispanic or Latino | 93 (70.5) | 43 (97.7) | 136 (77.3) |
| Hispanic or Latino | 23 (17.4) | 1 (2.3) | 24 (13.6) |
| Not reported | 16 (12.1) | 0 (0.0) | 16 (9.1) |
| **Weight, kg** |  |  |  |
| Mean (SD) | 82.4 (19.10) | 82.6 (12.72) | 82.45 (17.69) |
| Median (min, max) | 80.0 (51.0, 171.0) | 83.45 (56.3, 107.0) | 80.0 (51.0, 171.0) |
| **BMI, kg/m^2^** |  |  |  |
| Mean (SD) | 28.0 (5.63) | 27.9 (4.07) | 27.9 (5.27) |
| Median (min, max) | 27.15 (17.3, 57.1) | 27.3 (21.3, 39.1) | 27.2 (17.3, 57.1) |
| **LBM, kg** |  |  |  |
| Mean (SD) | 54.2 (9.75) | 55.1 (6.97) | 54.4 (9.12) |
| Median (min, max) | 52.9 (34.5, 79.6) | 55.4 (40.3, 66.0) | 53.8 (34.5, 79.6) |
| **IgG concentration, g/L^a^** |  |  |  |
| Mean (SD) | 14.8 (3.56) | 20.9 (6.22) | 16.5 (5.16) |
| Median (min, max) | 14.9 (6.6, 28.7) | 20.2 (11.6, 37.0) | 15.5 (6.6, 37.0) |
| **Total monthly dose, g** |  |  |  |
| Mean (SD) | 91.6 (50.94) | 96.6 (38.43) | 92.8 (48.07) |
| Median (min, max) | 86.5 (23.6, 266.4) | 83.6 (22.5, 175.5) | 86.2 (22.5, 266.4) |
| **CrCL, mL/min** |  |  |  |
| Mean (SD) | 115.5 (41.88) | 117.9 (29.34) | 116.1 (39.06) |
| Median (min, max) | 108.3 (53.9, 300.4) | 117.5 (64.2, 195.5) | 111.7 (53.9, 300.4) |
| **Treatment interval, *n* (%)** |  |  |  |
| Q2W | 2 (1.5) | 8 (18.2) | 10 (5.7) |
| Q3W | 14 (10.6) | 9 (20.5) | 23 (13.1) |
| Q4W | 116 (87.9) | 27 (61.4) | 143 (81.3) |

BMI, body mass index; CIDP, chronic inflammatory demyelinating polyradiculoneuropathy; CrCL, creatinine clearance; IgG, immunoglobulin G; LBM, lean body mass; max, maximum; min, minimum; MMN, multifocal motor neuropathy; Q2W, every 2 weeks; Q3W, every 3 weeks; Q4W, every 4 weeks; SD, standard deviation.

^a^Study 1: *n* = 122.

**SUPPLEMENTARY TABLE S2**	Parameter estimates and standard errors of the base popPK–PD model.

| **Parameter** | | **Final parameter estimate** | | **Magnitude of  variability** | |
| --- | --- | --- | --- | --- | --- |
|  |  | **Population mean** | **% RSE** | **Final estimate (% CV)** | **% RSE** |
| B | Logit of probability of increased adjusted INCAT score | 1.45 | 0.475 | NE | NA |
| BLKRel | Baseline response decrease rate | 0.100 | Fixed | NE | NA |
| KRin | Response maintenance rate | 0.00639 | 0.149 | NE | NA |
| E_max_ | Maximum effect on response decrease | 1.00 | Fixed | NE | NA |
| C50 | Change in IgG concentration^a^ of half-maximum effect, g/L | 0.120 | 0.117 | 1.05 (136) | 0.355 |
| Minimum value of the objective function = 295.581 | | | | | |

% CV, coefficient of variation expressed as a percentage; IgG, immunoglobulin G; INCAT, Inflammatory Neuropathy Cause and Treatment; NA, not applicable; NE, not estimated; popPK–PD, population pharmacokinetic–pharmacodynamic; % RSE, relative standard error expressed as a percentage.

^a^Change in IgG concentration from baseline.

Shrinkage estimates: 17.1% for overall change in IgG concentration of half-maximum effect.
